# Glucose Deprivation Induces G2/M Transition-Arrest and Cell Death in *N*-GlcNAc_2_-Modified Protein-Producing Renal Carcinoma Cells

**DOI:** 10.1371/journal.pone.0096168

**Published:** 2014-05-05

**Authors:** Takahiro Isono, Tokuhiro Chano, Asuka Kitamura, Takeshi Yuasa

**Affiliations:** 1 Central Reseach Laboratory, Shiga University of Medical Science, Otsu, Shiga, Japan; 2 Departments of Clinical Laboratory Medicine, Shiga University of Medical Science, Otsu, Shiga, Japan; 3 Departments of Urology, Cancer Institute Hospital, Japanese Foundation for Cancer Research, Tokyo, Japan; Georgia Regents University, United States of America

## Abstract

Some cancer cells can survive under glucose deprivation within the microenvironment of a tumor. Recently, we reported that *N*-linked (β-*N*-acetylglucosamine)_2_ [*N*-GlcNAc_2_]-modified proteins induce G2/M arrest and cell death under glucose deprivation. Here, we investigated whether such a response to glucose deprivation contributes to the survival of renal cell carcinomas, which are sensitive to nutritional stress. Specifically, we analyzed seven renal carcinoma cell lines. Four of these cell lines produced *N*-GlcNAc_2_-modified proteins and led G2/M-phase arrest under glucose deprivation, leading to cell death. The remaining three cell lines did not produce *N*-GlcNAc_2_-modified proteins and undergo G1/S-phase arrest under glucose deprivation, leading to survival. The four dead cell lines displayed significant up-regulation in the UDP-GlcNAc biosynthesis pathway as well as increased phosphorylation of p53, which was not observed in the surviving three cell lines. In addition, the four dead cell lines showed prolonged up-regulated expression of *ATF3*, which is related to unfolded protein response (UPR), while the surviving three cell lines showed only transient up-regulation of *ATF3*. In this study, we demonstrated that the renal carcinoma cells which accumulate *N*-GlcNAc_2_-modified proteins under glucose deprivation do not survive with abnormaly prolonged UPR pathway. By contrast, renal carcinoma cells that do not accumulate *N*-GlcNAc_2_-modified proteins under these conditions survive. Morover, we demonstrated that buformin, a UPR inhibitor, efficiently reduced cell survival under conditions of glucose deprivation for both sensitive and resistant phenotypes. Further studies to clarify these findings will lead to the development of novel chemotherapeutic treatments for renal cancer.

## Introduction

Cancer cells show an increase of glucose incorporation and utilization of glycolysis instead of mitochondrial respiration, even in the presence of oxygen. This phenomenon is called the Warburg Effect [1]. Therefore, many cancer cells are sensitive to glucose deprivation, which induces cell death [2]. However, some cancer cells can survive under glucose deprivation within the tumor microenvironment [3]. It is not known why there is this differential response among cancer cells to glucose deprivation. Nonetheless, we believe this difference in sensitivity to glucose deprivation may be important in the development of novel chemotherapeutic treatments.

Recently, we have demonstrated that *N*-linked (β-*N*-acetylglucosamine)_2_ [*N*-GlcNAc_2_]-modified glycoproteins are also induced under glucose deprivation in some cancer cells [4]. In these cancer cells, *N*-GlcNAc_2_-modified proteins produced under glucose deprivation promote the unfolded protein response (UPR) in the ER, which then induces G2/M arrest, leading to mitotic catastrophe [5].

We hypothesized that cancer cells producing *N*-GlcNAc_2_-modified proteins die under glucose deprivation. Here, we examined seven renal carcinoma cell lines that are resistant to conventional chemotherapeutic agents and are sensitive to nutritional starvation [6]. Specifically, we investigated whether these renal carcinoma cells produce *N*-GlcNAc_2_-modified proteins under glucose deprivation. Our results show that four of these cell lines produce *N*-GlcNAc_2_-modified proteins, arrest at G2/M-phase and finally die. The remaining three cell lines did not produce *N*-GlcNAc_2_-modified proteins and survived under conditions of glucose deprivation.

## Materials and Methods

### Cell lines and cell culture conditions

We used seven renal cell carcinoma cell lines in this study (Caki1, Caki2, NC65, ACHN, SW839, VMRC-RCW and KMRC-1). These cell lines were purchased from either American Type Culture Collection, Riken Cell Bank, Cell Resource Center for Biomedical Research in Tohoku University or the Japanese Collection of Research Bioresources. All the cell lines were maintained in RPMI 1640 (Life Technologies, Carlsbad, CA), which contained 25 mM glucose, supplemented with 10% fetal calf serum (FCS), penicillin (100 U/ml) and streptomycin (100 µg/ml) at 37°C in a humidified 5% CO_2_ atmosphere. In the experimental culture, cells were seeded in high-glucose medium and then the culture medium was replaced on day 2 with fresh high-glucose medium (25 mM glucose) or with glucose deprivation medium, which was depleted of glucose (0 mM glucose, Life Technologies) after two times washing with glucose deprivation medium. Treatment with PUGNAc (100 µM), an inhibitor of β-D-*N*-acetylglucosaminase (*O*-GlcNAcase), buformin (10–100 µM), an inhibitor of UPR, or temsirolimus (50 nM), an inhibitor of mTOR, azaserine (50 µM), an inhibitor of hexosamine biosynthetic pathway, or tunicamycin (2 µg/ml), an inhibitor of *N*-glycosylation, was carried out when the medium was replaced. Cells on day 2–6 were used for the experiments i.e., cell samples were collected 0–4 days after replacement with fresh medium on day 2.

### Antibodies

Anti-*O*-GlcNAc (#MMS-248R, CTD110.6) IgM monoclonal antibody was purchased from Covance (Berkeley, CA). Anti-p53 (DO-1) (sc-126) and anti-p21 (sc-6246) monoclonal antibodies were purchased from Santa Cruz Biotechnology (Santa Cruz, CA). Anti-phospho-p53 (Ser15) (#9284) and anti-GADD45A (#4632) antibodies were purchased from Cell Signaling Technology (Beverly, MA). Anti-RB1 (G3-245) and anti-BiP (clone 40) monoclonal antibodies were purchased from BD Transduction Laboratories (Franklin Lakes, NJ). Anti-α-tubulin (#T9026, DM1A) monoclonal antibodies were purchased from Sigma-Aldrich (St. Louis, MO).

### Immunoblotting

Cells were lysed in Laemmli-SDS buffer, subjected to SDS-polyacrylamide gel electrophoresis, and electro-transferred to membrane filters (Immuno-Blot PVDF membranes, Bio-Rad Laboratories, Richmond, CA). The filters were incubated with a primary antibody in TBS-T containing 2% bovine serum albumin (BSA) overnight and incubated for 1 hour in horseradish peroxidase-conjugated anti-mouse, anti-rabbit (Cell Signaling Technology), or anti-mouse IgM (Sigma-Aldrich) diluted 1∶5,000 in TBS-T containing 2% BSA. Immunoreactivity was detected using the ECL system (GE Healthcare) with LAS4000 (Fujifilm, Tokyo, Japan) and quantified with Multi gauge (Fujifilm), using an anti-α-tubulin antibody as an internal control.

### Cell cycle analysis

Treated cells were harvested with the cultured medium and washed in cold PBS before being fixed with 1% buffered formalin and cold 70% ethanol. The cells were permeabilized and incubated with anti-MPM-2 (diluted 1∶500) (FOXM1, 0.T.181; Abcam, Cambridge, UK) overnight, followed by treatment with Alexa 488 goat anti-mouse IgG (H+L) (Life Technologies). The samples were then washed in cold PBS and transferred to tubes containing 1 µg/ml FxCycle Violet (Life Technologies). Cells labeled with MPM-2, which is characteristic of early M-phase, and at various phases of the cell cycle were analyzed on an Attune Acoustic Focusing Cytometer and the cytometric software (Life Technologies).

### Cell Viability

4×10^4^ cells were plated onto 24 well culture plates and cultured at 37°C. For the cell viability assay, cells were collected 0–4 days after the fresh medium replacement on day 2. The percentage of dead and living cells was then determined by staining with 0.1% trypan blue.

### Quantitative reverse-transcription–polymerase chain reaction (qRT-PCR)

Total RNA from renal cell carcinoma cells was extracted using acid guanidinium thiocyanate-phenol-chloroform [7]. Quantitative RT-PCR was performed using the LightCycler 480 SYBG Master I Mix and LightCycler 480 System II (Roche Diagnostics, Mannheim, Germany). Gene expression was normalized using the *GAPDH* gene. Primer sequences are provided in Table S1. All quantification analyses were performed in triplicate.

### Stastitics

Results of experiments are represented as mean ± S.E. Each mean represents data from at least three independent experiments. The Student's *t* test (two-tail) was used to compare differences between groups.

## Results

### The production of *N*-GlcNAc_2_-modified proteins in renal carcinoma cells under glucose deprivation

**Figure 1:**
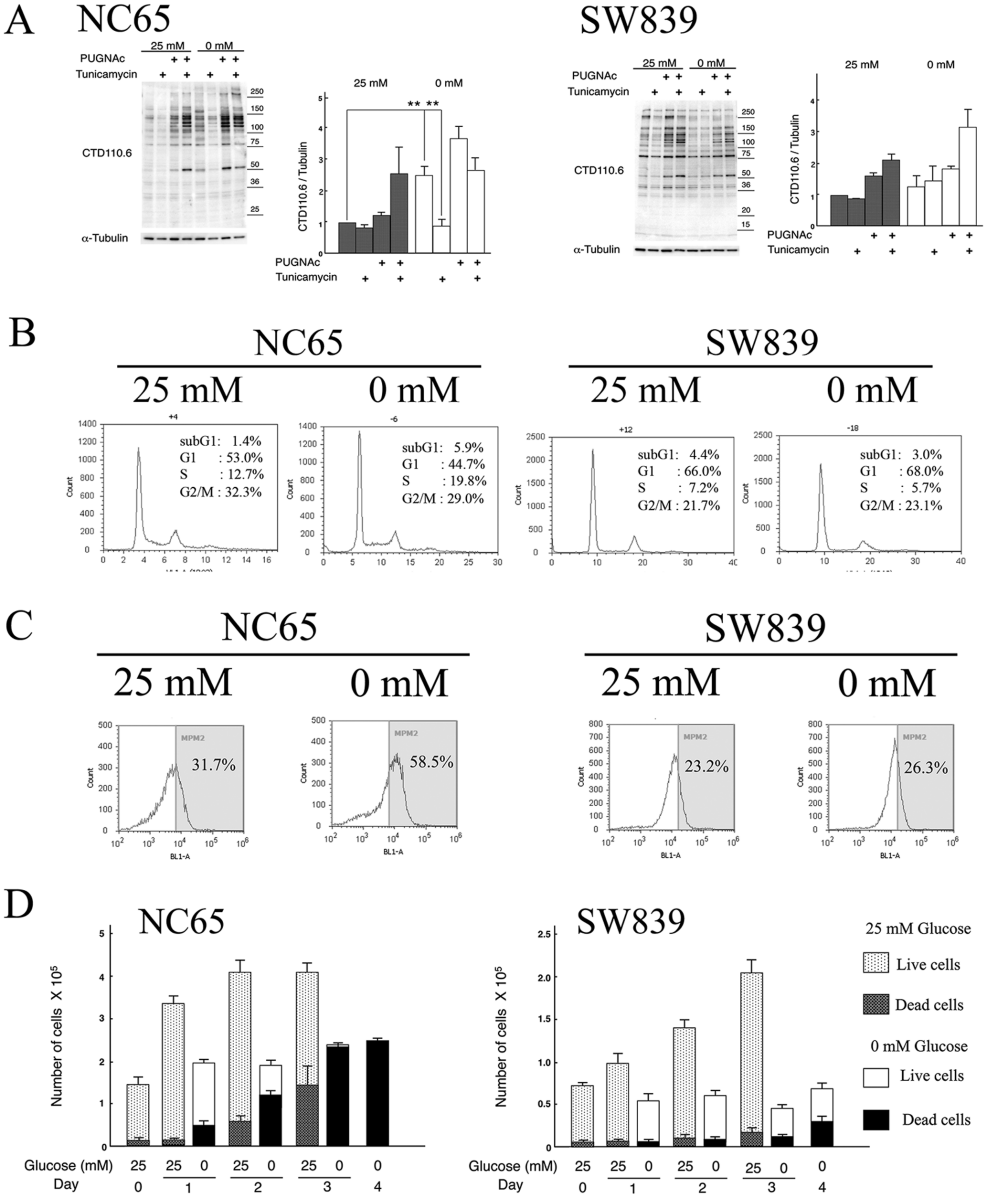
Characterization of NC65 and SW839 cells. **A**, Immunoblot analysis. NC65 and SW839 cells were seeded in high-glucose medium and then the culture medium was replaced on day 2 with fresh high-glucose medium (25 mM glucose) or with glucose-deprived medium (0 mM glucose) for 24 h. Treatment with PUGNAc (100 µM), an inhibitor of β-D-*N*-acetylglucosaminase (*O*-GlcNAcase), and tunicamycin (2 µg/ml), an inhibitor of *N*-glycosylation, were carried out on when the medium was replaced. For each cell type: left panel, immunoblot using CTD110.6 and anti-α-tubulin antibodies; right panel, quantitative analysis of reactivity with the CTD110.6 antibody, normalized against the anti-α-tubulin signal for untreated cells grown in high-glucose medium (25 mM glucose). An anti-α-tubulin antibody was used as an internal control. Error bars represent standard error from three independent experiments. ** represents p<0.01. **B-C**, Flow cytometric analysis. NC65 and SW839 cells were cultured in fresh 25 mM or 0 mM glucose medium for 24 h after 2 days of culture in 25 mM glucose medium and then fixed and stained using FxCycle Violet nuclear staining reagent (**B**) and anti-MPM-2 antibody (**C**). **D**, Cell growth. The numbers of living and dead cells were counted using the trypan-blue exclusion assay on 0–4 days after the fresh medium replacement on day 2. Note that glucose deprivation of NC65 cells induced the production of *N*-GlcNAc_2_-modified proteins and G2/M transition arrest, leading to cell death. However, glucose deprivation of SW839 cells did not induce the production of *N*-GlcNAc_2_-modified proteins, and induced G1/S transition arrest, thereby allowing cell survival. Glucose deprivation also enhanced the level of MPM-2 in NC65 cells, but did not in SW839 cells.

An *O*-GlcNAc-specific antibody (CTD110.6) was utilized to detect *N*-GlcNAc_2_-modified proteins in renal cell carcinoma cells under glucose deprivation. Detection of *N*-GlcNAc_2_-modified proteins was assessed by tunicamycin-sensitive cross-reactivity to CTD110.6 antibody in an immunoblot analysis [4]. Using this procedure, four out of the seven renal cell carcinoma cell lines (NC65, ACHN, Caki1 and Caki2) were judged to produce *N*-GlcNAc_2_-modified proteins. These cell lines displayed significantly elevated levels of proteins that cross-reacted with CTD110.6 antibodies when incubated in the absence of glucose (0 mM) compared to incubation in the presence of glucose (25 mM). Moreover, this increased cross-reactivity with CTD110.6 antibodies was significantly reduced after treatment with tunicamycin (Figure 1A and Figure S1A), suggesting that much of the signal was derived from *N*-GlcNAc_2_-modified proteins. The other three renal cell carcinoma cell lines (SW839, VMRC-RCW and KMRC-1) did not produce *N*-GlcNAc_2_-modified proteins. Specifically, these cell lines showed no change in cross-reactivity with CTD110.6 antibodies in the absence or presence of glucose (Figure 1A and Figure S1A). We also analyzed the production of *O*-GlcNAc_2_-modified proteins induced by PUGNAc [4]. However, no differences were observed among these seven cell lines.

### Different types of cell cycle arrest among renal cell carcinoma cells under glucose deprivation

Flow cytometry was used to evaluate the cell cycle phases of renal carcinoma cells maintained under conditions of glucose deprivation (Figure 1B and Table S2). In the three cell lines that did not produce *N*-GlcNAc_2_-modified proteins, we found a decrease of S-phase cells with a concomitant increase in G1-phase cells under glucose deprivation for 24 h. These results showed that glucose deprivation induced G1/S transition arrest in cell lines that did not produce *N*-GlcNAc_2_-modified proteins. By contrast, we found a decrease in the number of G1-phase cells with a concomitant increase in S- and subG1-phase cells under glucose deprivation at 24 h in the four cell lines producing *N*-GlcNAc_2_-modified proteins. The number of cells expressing the early M-phase marker, MPM-2 (FOXM1) [8], also increased during glucose deprivation (Figure 1C and Table S2). These results showed that glucose deprivation induced the cell death on early M-phase with the transition of G1- and S-phase in cell lines producing *N*-GlcNAc_2_-modified proteins. A kind of G2/M arrest, leading to mitotic catastrophe as described previously [5], would be induced by glucose deprivation.

### Cell viability in renal cell carcinoma cells is associated with the production of *N*-GlcNAc_2_-modified proteins during glucose deprivation

The cell viability of renal carcinoma cells under glucose deprivation was evaluated using the trypan-blue exclusion assay (Figure 1D and Figure S2). In the four cell lines producing *N*-GlcNAc_2_-modified proteins, glucose deprivation for 1 day stopped cell growth and 10–30% of the cells died. After 3–4 days of glucose deprivation, very few of the cells were viable. However, subjecting the three cell lines that do not produce *N*-GlcNAc_2_-modified proteins to glucose deprivation for 1 day stopped cell growth, but less than 10% of the cells were dead. On day 4 of glucose deprivation, about half of the cells were alive. Indeed, most of these cells remained viable after more than 2 weeks of glucose deprivation. Moreover, cell growth resumed when these surviving cells were transferred to the culture medium containing glucose. Subtlely heterogenic components among SW839 cells, which produced some *N*-GlcNAc_2_-modified proteins, were subject to cell death under prolonged glucose deprivation. On the other side, NC65 cell growth resumed when surviving cells, which showed a reduction of *N*-GlcNAc_2_-modified proteins (Figure S1B), were transferred to fresh culture medium containing glucose. Thus, under conditions of prolonged glucose deprivation, the presence or absence of *N*-GlcNAc_2_-modified proteins strictly correlates with cell death or survival, respectively.

### *N*-GlcNAc_2_-modified proteins correlate with activation of the UDP-GlcNAc biosynthesis-pathway in renal cell carcinomas under glucose deprivation

**Figure 2:**
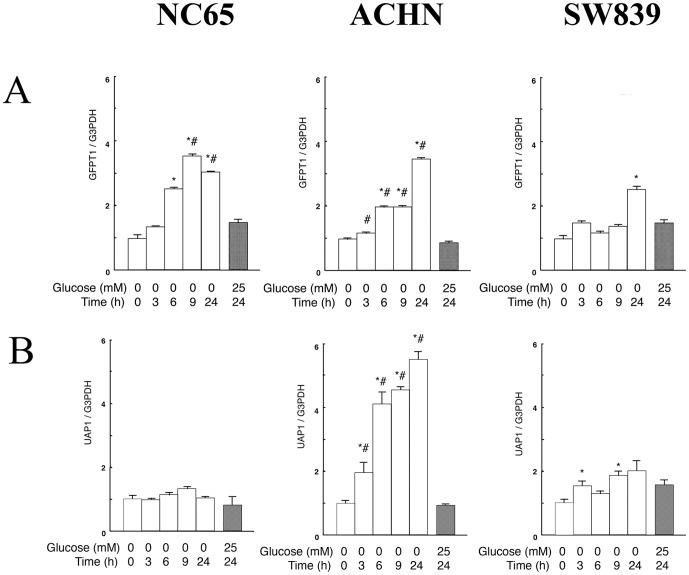
Quantitative RT-PCR of *GFPT1* and *UAP1* belonging to the UDP-GlcNAc biosynthesis-pathway in NC65, ACHN and SW839 cells. Quantitative RT-PCR of *GFPT1* (A) and *UPA1* (B) was performed on NC65, ACHN and SW839 cells. The cells were either incubated in 25 mM or 0 mM glucose medium. Gene expression was normalized against *GAPDH* transcripts. Error bars represent standard errors from three independent experiments. * and #: signify p<0.05 against 0 mM glucose at 0 h and 25 mM glucose at 24 h, respectively. Note that in renal carcinoma cells producing *N*-GlcNAc_2_-modified proteins (NC65 and ACHN) the expression of *GFPT1* or *UAP1*, associated with the UDP-GlcNAc biosynthesis pathway, was significantly activated (by approximately 3-fold) 6–9 h after glucose deprivation. However, no such activation was observed in renal carcinoma cells that do not produce *N*-GlcNAc_2_-modified proteins (SW839).

In a recent study, we showed that T24 cells producing *N*-GlcNAc_2_-modified proteins under glucose deprivation activated the UDP-GlcNAc biosynthesis-pathway and UPR together with concomitant repression of G2/M transition related genes [5]. Here, we evaluated three different factors; namely the UDP-GlcNAc biosynthesis-pathway, cell cycle molecules (including G2/M transition genes) and UPR. In the type of renal carcinoma cells producing *N*-GlcNAc_2_-modified proteins, the expression of *GFPT1* (glutamine-fructose-6-phosphate aminotransferase 1), a rate-limiting enzyme belonging to the UDP-GlcNAc biosynthesis pathway, or *UAP1* (UDP-N-acetylglucosamine pyrophosphorylase 1), a key enzyme belonging to the UDP-GlcNAc biosynthesis pathway, was significantly activated (i.e., 3-fold increase) 6–9 h after the start of glucose deprivation. By contrast, in renal carcinoma cells that do not produce *N*-GlcNAc_2_-modified proteins, *GFPT1* and *UAP1* were less activated (i.e.,<2-fold increase) by glucose deprivation over the same timescale (Figure 2 and Table S3). These results strongly suggested that the production of *N*-GlcNAc_2_-modified proteins correlates with activation of the UDP-GlcNAc biosynthesis-pathway in renal cell carcinoma cells under glucose deprivation.

### The differences of cell cycle molecules in two types of renal cell carcinomas under glucose deprivation

**Figure 3:**
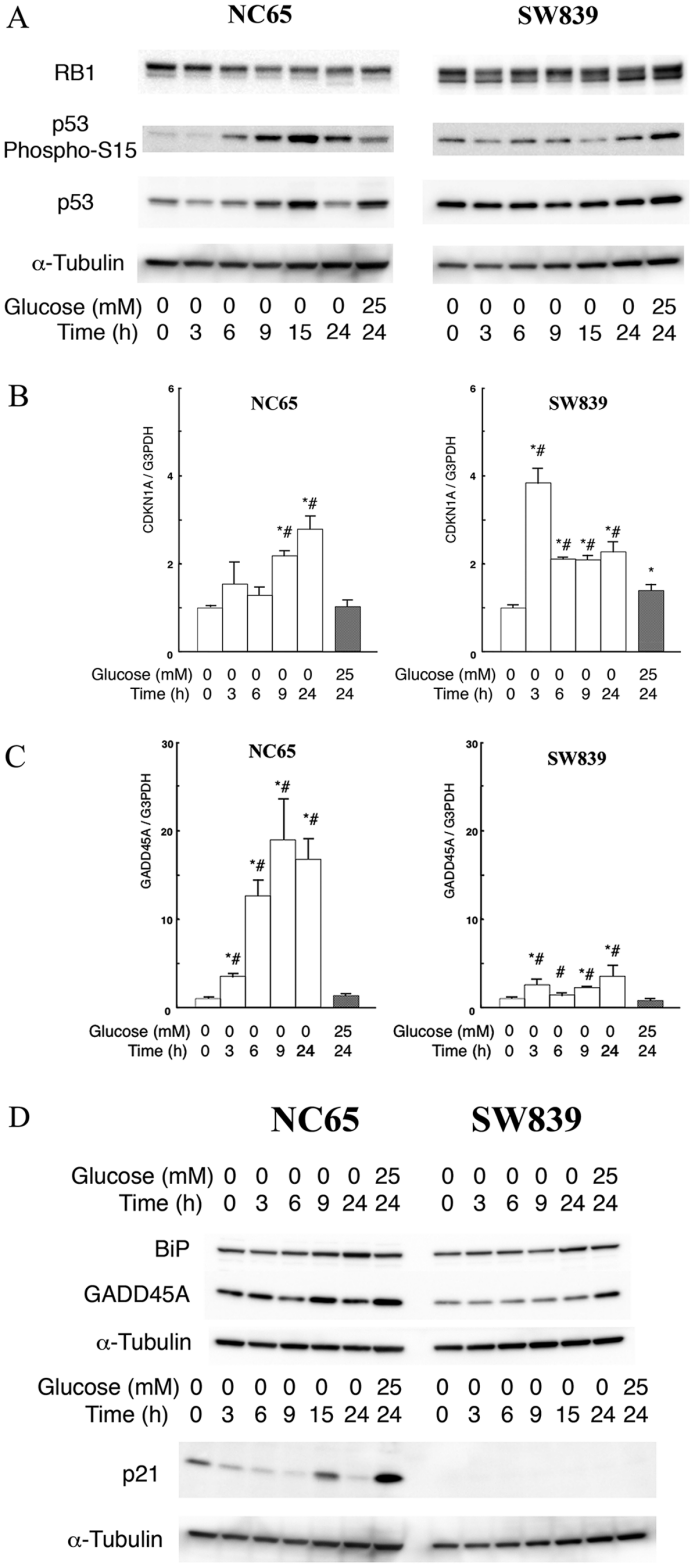
The differences of cell cycle molecules in two types of renal cell carcinomas under glucose deprivation. **A**, Immunoblot analysis. The panels show immunoblots for RB1, S15-phosphorylated p53, total p53 and α-tubulin. **B–C**, Quantitative RT-PCR data of *CDKN1A* (**B**) and *GADD45A* (**C**) for NC65 and SW839 cells. The cells were either incubated in 25 mM or 0 mM glucose medium. Gene expression was normalized against *GAPDH* transcripts. Error bars represent standard errors from three independent experiments. * and #: signify p<0.05 against 0 mM glucose at 0 h and 25 mM glucose at 24 h, respectively. Note that the expression of S15-phosphorylated p53 and the expression of *GADD45A* significantly increased under glucose deprivation in NC65 cells compared with SW839 cells. **D**, Immunoblots for BiP, GADD45A, p21/CDKN1A and α-tubulin in NC65 SW836 cells. Note that glucose deprivation increased the level of BiP and GADD45A in NC65 cells.

The expression of mitotic kinase genes associated with the G2/M transition was examined by qRT-PCR in renal carcinoma cells under glucose deprivation. Our results show that these genes were suppressed to the same extent in both cell types associated with *N*-GlcNAc_2_-modified proteins (data not shown). We confirmed the cell cycle status of the renal cell carcinoma cells by performing immunoblots for RB1 and S15-phosphorylated p53. In the two types of cells with or without production of *N*-GlcNAc_2_-modified proteins, total RB1 and phosphorylated RB1 (pRB1) slowly decreased 15 h after the start of glucose deprivation. However, no significant differences were observed between the two cell types (Figure 3A, Figure S3A and S3B). These results indicated that G1/S arrest caused by RB1 regulation did not occur 3–9 h after initiating glucose deprivation in any of the renal carcinoma cell lines. However, the level of S15-phosphorylated p53 significantly increased under glucose deprivation only in the cell types producing *N*-GlcNAc_2_-modified proteins (Figure 3A, Figure S3A and S3C). Activation of p53 can affect both G1/S and G2/M arrest [9]. Therefore, we used qRT-PCR to examine the expression of two effectors of p53: *CDKN1A*, which acts to arrest the G1/S transition, and *GADD45A*, which acts to arrest the G2/M transition (Figure 3B and 3C, Table S4). In the cell type producing *N*-GlcNAc_2_-modified proteins, the expression of *GADD45A* increased 20-fold under glucose deprivation, while the expression level of *CDKN1A* showed only a moderate increase (<4-fold). Our observations suggested that G2/M arrest in these cells was primarily caused by p53 activation. However, when the other type of cells that do not produce *N*-GlcNAc_2_-modified proteins were subjected to glucose deprivation, the expression of both *GADD45A* and *CDKN1A* increased by less than 4-fold. These results suggest that the specific phase of cell cycle arrest was not enhanced, but the cell cycle might reduce globally under glucose deprivation. Immunoblot analysis for GADD45A and CDKN1A in NC65 and SW839 cells support the transcriptional differences, although the observed increase of protein expression was less than that of the corresponding increase in transcription (Figure 3D). In the expressional differences between *GADD45A* and *CDKN1A*, the other factors may contibute to the regulation of these gene expressions and/or to p53 phospholylation of another residues except S15 site.

### Differences between the two types of renal cell carcinomas under glucose deprivation in terms of UPR and modified cell death after treatment with Buformin

**Figure 4:**
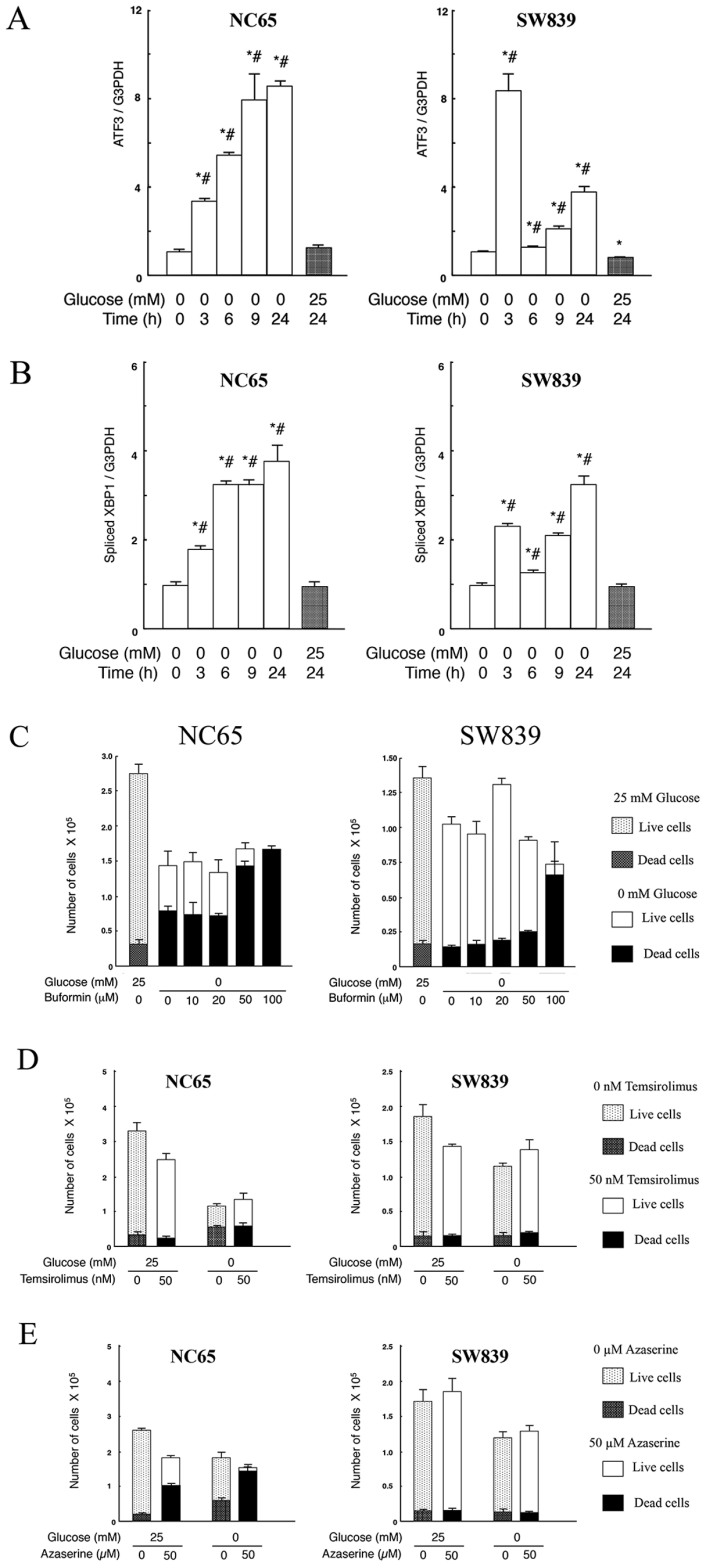
The differences of UPR and modified cell death induced by Buformin in two types of renal cell carcinomas under glucose deprivation. A–B, Quantitative RT-PCR of *ATF3* (A) and spliced *XBP1* (B) was performed on NC65 and SW839 cells. The cells were either incubated in 25 mM or 0 mM glucose medium. Gene expression was normalized against *GAPDH* transcripts. Error bars represent standard errors from three independent experiments. * and #: signify p<0.05 against 0 mM glucose at 0 h and 25 mM glucose at 24 h, respectively. C–E, NC65 and SW839 cells were cultured in 25 mM or 0 mM glucose medium with or without buformin (C) or temsirolimus (D) or azaserine (E) for 24 h. The numbers of living and dead cells were counted using the trypan-blue exclusion assay. Note that for cell types producing *N*-GlcNAc_2_-modified proteins (NC65), the expression of *ATF3* and spliced *XBP1* showed a significant and continuous increase during glucose deprivation. By contrast, in cell types not producing *N*-GlcNAc_2_-modified proteins (SW839), *ATF3* and spliced *XBP1* were transitionally activated 3 h after intitiating glucose deprivation but did not increase any further. NC65 cells died after incubation with 50 µM buformin. SW839 cells underwent significant cell death following incubation with 100 µM buformin. Temsirolimus did not induce significant levels of cell death in NC65 and SW839 cells grown in either medium. Azaserine did induce substantial levels of cell death in NC65 cells grown in the absence or presence of glucose, although it did not induce cell death in SW839 cells.

Finally, we evaluated UPR related genes in renal cell carcinoma cells under glucose deprivation. Specifically, we investigated the expression of *ATF3*, a transcription factor belonging to UPR related genes. In the cell type producing *N*-GlcNAc_2_-modified proteins the expression of *ATF3* showed a marked and continuous increase during glucose deprivation. By contrast, analysis of cells that did not produce *N*-GlcNAc_2_-modified proteins showed *ATF3* to be transiently activated 3 h after glucose deprivation, but this up-regulation was not prolonged (Figure 4A and Table S5). Moreover, analysis of *XBP1* splicing and BiP/GRP78 protein expression as UPR markers showed that cell types with *N*-GlcNAc_2_-modified proteins displayed a more striking increase in the level of these markers than cell types lacking *N*-GlcNAc_2_-modified proteins (Figure 4B, Table S5, and Figure 3D). These results indicated that UPR was induced in both cell types with and without *N*-GlcNAc_2_-modified proteins, but that there were some differences of signaling. Significant and continuous enhancment in UPR is associated with cell death in renal carcinomas producing *N*-GlcNAc_2_-modified proteins. However, transient UPR is associated with cell survival in renal carcinomas lacking *N*-GlcNAc_2_-modified proteins.

We also examined the effect of buformin, a biguanide and potential antitumorigenic agent that inhibits UPR [10], [11], on renal cell carcinomas under glucose deprivation. Buformin (100 µM, 1 day) induced complete cell death in renal cell carcinomas without *N*-GlcNAc_2_-modified proteins under glucose deprivation. Corresponding treatment with buformin also facilitated cell death in the renal cell carcinomas that produce *N*-GlcNAc_2_-modified proteins (Figure 4C and Figure S4). Cell death caused by buformin was not accompanied by additional *N*-GlcNAc_2_-modified proteins (data not shown). Our findings indicate that buformin is an effective antitumorigenic agent for all types of renal cell carcinomas regardless of whether the cells produce *N*-GlcNAc_2_-modified proteins.

Moreover, we also examined the effect of temsirolimus, an inhibitor of mTOR [14], and azaserine, an inhibitor of GFPT1 in the hexosamine biosynthetic pathway [15], (Figure 4D and E). Neither of these inhibitors caused cell death in renal carcinomas lacking *N*-GlcNAc_2_-modified proteins under glucose deprivation. However, azaserine did induce cell death in renal carcinomas producing *N*-GlcNAc_2_-modified proteins in culture medium with or without glucose.

## Discussion

Here, we demonstrate that there are two types of renal carcinomas that are “sensitive” and “resistant” to glucose deprivation. The general characteristics of the “sensitive” and “resistant” type of renal cell carcinomas under glucose deprivation are summarized in Table 1. Our study showed that NC65, ACHN, Caki1 and Caki2 cells were “sensitive” type and SW839, VMRC-RCW and KMRC-1 were of the “resistant” type. However, akin to KMRC-1 cells that do not fulfill all the characteristics of either group, there may be rare cases of intermediate cell types that fall between the “sensitive” and “resistant” phenotype. Glucose deprivation in the “sensitive” type of renal cell carcinomas induces the production of *N*-GlcNAc_2_-modified proteins and G2/M transition arrest prior to cell death. These type of cells are characterized by both continuous activation of UPR and mitotic catastrophic death [5]. By contrast, the “resistant” type of renal cell carcinomas do not produce *N*-GlcNAc_2_-modified proteins, and do not undergo G2/M transition arrest under conditions of glucose deprivation; and subsequently arrested at G1 phase and survive. These differences between the cell types depend on carbon metabolism and cell signaling under glucose deprivation, which will influence the cellular response to nutrient starvation. Given that glucose deprivation is an important mechanism of the current targeted therapy for nutritional starvation of renal cell carcinomas, any differences in carbon metabolism and/or cell signaling between these two types of renal cell carcinomas might be key factors in determining the efficacy of this targeted therapy. Therefore, our findings may account for the chemotherapeutic resistance of some types of renal carcinomas, and may lead to the development of effective novel drug combinations.

**Table 1:** Summary of differences in “sensitive” and “resistant” type of renal cell carcinomas under glucose deprivation.

	“sensitive” type	“resistant” type
*N*-GlcNAc_2_	Produced	No
Cell cycle	G2/M arrest	G1/S arrest
Glucose Deprivation	Cell death	Survival
UDP-GlcNAc synthesis	Activated	No
Phosph-S15 p53	Increased	No
GADD45A	Significant increase	No
ATF3	Strongly and continuously activated	Weakly and transiently activated

GFPT1, which is a limiting enzyme in the UDP-GlcNAc biosynthesis pathway, was significantly up-regulated in bladder caricinoma cells producing *N*-GlcNAc_2_-modified proteins during glucose deprivation [5]. By contrast, other enzymes that utilize fructose-6-P as a substrate, which belong to the glycolysis, gluconeogenesis and GDP-mannose synthesis pathways, displayed reduced levels of expression in these cells under the same conditions [5]. The data suggested that glucose metabolism under glucose deprivation was redirected to the synthesis of UDP-GlcNAc, which is a substrate for *N*-GlcNAc_2_-modification. Therefore, the production of *N*-GlcNAc_2_-modified proteins will be influenced not only by the UDP-GlcNAc biosynthesis pathway but also by the other pathways related to glucose metabolism and cell death. G2/M transition-arrest accompanied the reduction of G2/M transition-related genes and/or the complex induction of G2/M arrest-related genes such as *GADD45A* and *GADD45B* [5]. The cellular characteristics of “sensitive” and “resistant” types of renal cell carcinomas may be controlled by unknown sets of genes that modify expression of *GFPT1*, *UPA1*, *CDKN1A*, *GADD45A* and *ATF3*. Global transcriptome analyses using next genaration sequencers will help to resolve these issues.

The “resistant” type of renal cell carcinomas could survive indefinitely under glucose deprivation. Inhibitors of mammalian targets of rapamycin (mTOR) or vascular endothelial growth factor receptor (VEGFR) will be applied clinically for the treatment of renal cell carcinomas with the aim of depriving the cells of nutrition [12], [13]. However, our findings suggest these agents will not be effective against the “resistant” type of renal cell carcinomas. Our studies showed that temsirolimus, an inhibitor of mTOR [14], reduced cancer cell growth slightly, but did not cause cell death against the “resistant” type of renal cell carcinomas under glucose deprivation (Figure 4D). In the present study, buformin, a biguanide used in the treatment of diabetes mellitus, was found to effectively kill the “resistant” type of renal cell carcinomas that had survived glucose deprivation. Thus, buformin acted as an effective antitumorigenic agent. We propose that the inhibition of UPR by buformin induced cell death in the “resistant” type of renal cell carcinomas. Indeed, a previous report [11] indicated that the antitumor action of biguanides containing buformin could be mediated by 4E-BP1 hyperactivation, which results in UPR inhibition and selective cell killing when glucose is withdrawn. Inhibition of UPR by buformin may induce the death of all types of cancer cells. UPR is beneficial to various tumor cells, which increases protein folding capacity leading to a growth advantage, and UPR down-regulation allows the other cancer cells to escape the apoptotic pathway favoring tumorigenesis [16], [17].

The production of *N*-GlcNAc_2_-modified proteins may be a useful marker for renal cell carcinoma death under glucose deprivation. Various cancer cells produce *N*-GlcNAc_2_-modified proteins and subsequently die under glucose deprivation [4]. Thus, the detection of *N*-GlcNAc_2_-modified proteins can be a useful marker for evaluating the cancer microenvironment and facilitating strategies to induce cancer cell death. However, this detection must be judged by tunicamycin-sensitive cross-reactivity to CTD110.6 antibody using an immunoblot assay [4]. Therefore, a specific antibody against *N*-GlcNAc_2_ is required, which will be invaluable in assessing the treatment regime for these types of cancer.

The results shown in Figure 1D suggest that the “sensitive” type of renal cell carcinomas proliferate faster in high-glucose medium (25 mM glucose) by comparison to the “resistant” type of renal cell carcinomas. Our data with azaserine, an inhibitor of GFPT1, shown in Figure 4E, suggest that the “sensitive” type of renal cell carcinomas were also sensitive to azaserine in high-glucose medium (25 mM glucose), but this was not the case with the “resistant” type of renal cell carcinomas. These results suggest that there were metabolic differences between the “sensitive” and “resistant” type of cells in high-glucose medium, and that the original status of both types of renal cell carcinomas may be used to predict their fate under glucose deprivation. Therefore, these differences in the original status between the two types of renal cell carcinomas can act as predictive markers for assessing the likely efficacy of antitumorigenic agents under conditions of nutritional starvation. Global transcriptome analyses of the two types of renal cell carcinomas in high-glucose medium will contribute to the identification of such predictive biomarkers.

The present study demonstrates that the presence or absence of *N*-GlcNAc_2_-modified proteins correlate with death or survival of renal cell carcinomas under glucose deprivation, respectively. Glucose deprivation is an important mechanism for current therapeutic strategies used in the treatment of renal cell carcinomas. Our study has highlighted differences of carbon metabolism and cell signaling between two types of renal cell carcinomas that might be key factors in the efficacy of novel targeted therapies. Additional investigations are expected to further clarify this issue.

## Supporting Information

Figure S1 **Immunoblot analysis of renal cell carcinomas for CTD110.6 antibody.** A. Renal cell carcinomas were incubated in glucose-deprived medium (0 mM glucose) for 24 h. For each cell type: left panel, immunoblot using CTD110.6 and anti-α-tubulin antibodies; right panel, quantitative analysis of reactivity with the CTD110.6 antibody, normalized against the anti-α-tubulin signal for untreated cells grown in high-glucose medium (25 mM glucose). An anti-α-tubulin antibody was used as an internal control. Tunicamycin and PUGNAc are inhibitors of *N*-glycosylation and β-D-*N*-acetylglucosaminase (*O*-GlcNAcase), respectively. Error bars represent standard error from three independent experiments. * and ** represent p<0.05 and p<0.01, respectively. Note that ACHN, Caki1 and Caki2 highly produced *N*-GlcNAc_2_-modified proteins under glucose deprivation, whereas VMCR-RCW and KMRC-1 cells did not. B. Immunoblot for CTD110.6 and anti-α-tubulin antibodies. NC65 and SW839 cells were seeded in high-glucose medium and then the culture medium was replaced on day 2 with fresh high-glucose medium (25 mM glucose) or with glucose-deprived medium (0 mM glucose) for 1–2 days. Two days after replacement of medium, both sets of cells were transferred into fresh medium containing glucose.
(TIF)

Figure S2 **Cell growth under glucose deprivation in renal cell carcinomas.** The numbers of living and dead cells were counted using the trypan-blue exclusion assay. Note that glucose deprivation significantly induced cell death in ACHN, Caki1 and Caki2 cells, but not in VMCR-RCW and KMRC-1 cells.
(TIF)

Figure S3 **Immunoblot analysis of renal cell carcinomas.** **A**, Immunoblots for RB1, S15-phosphorylated p53, total p53 and α-tubulin. **B–C**, Quantitative analysis of reactivity with the phosphorylated RB1 (pRB1) (**B**, upper bands), normalized to the total signal, and the S15-phosphorylated p53 (**C**), normalized to the total p53 signal in 0 mM glucose and 0 h, respectively. * and #: signify p<0.05 against 0 mM glucose at 0 h and 25 mM glucose at 24 h, respectively. Note that glucose deprivation significantly reduced the level of phosphorylated RB1 in ACHN, Caki1 and Caki2 cells. S15-phosphorylated p53 was induced under conditions of glucose deprivation in NC65, ACHN, Caki1 and Caki2 cells.
(TIF)

Figure S4 **Buformin could induce cell death in all types of renal cell carcinomas under conditions of glucose deprivation.** Renal cell carcinomas were cultured in 25 mM or 0 mM glucose medium with or without buformin for 24 h. The numbers of living and dead cells were counted using the trypan-blue exclusion assay. Note that buformin could induce significant levels of cell death in all types of renal cell carcinomas under glucose deprivation.
(TIF)

Table S1 **Oligonucleotides used for qRT-PCR.**
(DOC)

Table S2 **Flow cytometric analysis for renal cell carcinomas.**
(DOC)

Table S3 **Quantitative RT-PCR data of** ***GFPT1*** **and** ***UAP1*** **belonging to the UDP-GlcNAc biosynthesis-pathway in renal cell carcinomas.**
(DOC)

Table S4 **Quantitative RT-PCR data of** ***GADD45A*** **and** ***CDKN1A*** **belonging to the p53 signaling-pathway in renal cell carcinomas.**
(DOC)

Table S5 **Quantitative RT-PCR data of** ***ATF3*** **and** ***XBP1*** **belonging to the UPR genes in renal cell carcinomas.**
(DOC)
