# Supplementary figures and images for: Glucose Deprivation Induces G2/M Transition-Arrest and Cell Death in N-GlcNAc2-Modified Protein-Producing Renal Carcinoma Cells
Source: PLoS One. 2014 May 5;9(5):e96168. doi: 10.1371/journal.pone.0096168 (PMC4010426; doi:10.1371/journal.pone.0096168)

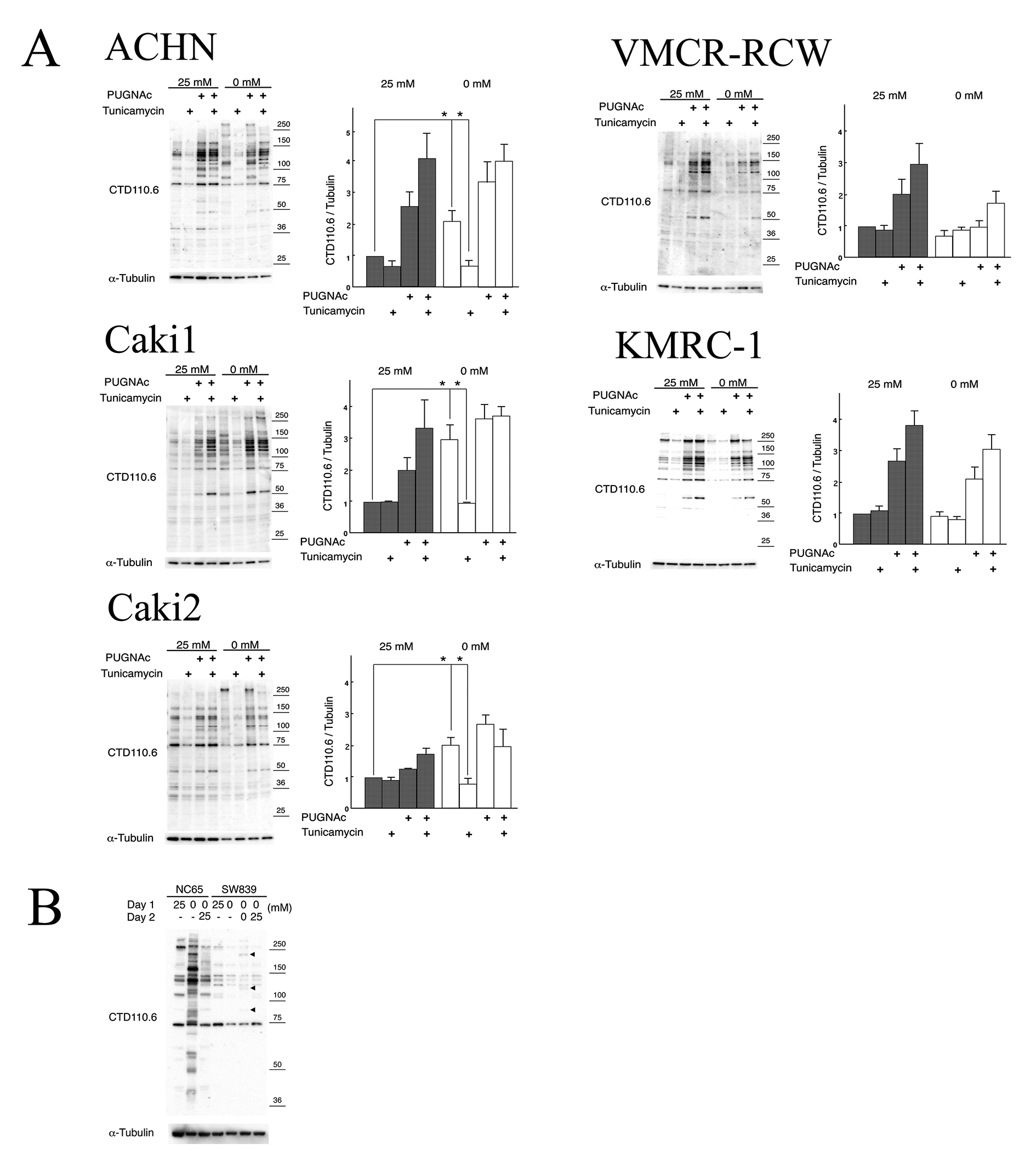

Supplement: Figure S1 — Immunoblot analysis of renal cell carcinomas for CTD110.6 antibody. A. Renal cell carcinomas were incubated in glucose-deprived medium (0 mM glucose) for 24 h. For each cell type: left panel, immunoblot using CTD110.6 and anti-α-tubulin antibodies; right panel, quantitative analysis of reactivity with the CTD110.6 antibody, normalized against the anti-α-tubulin signal for untreated cells grown in high-glucose medium (25 mM glucose). An anti-α-tubulin antibody was used as an internal control. Tunicamycin and PUGNAc are inhibitors of N-glycosylation and β-D-N-acetylglucosaminase (O-GlcNAcase), respectively. Error bars represent standard error from three independent experiments. * and ** represent p<0.05 and p<0.01, respectively. Note that ACHN, Caki1 and Caki2 highly produced N-GlcNAc2-modified proteins under glucose deprivation, whereas VMCR-RCW and KMRC-1 cells did not. B. Immunoblot for CTD110.6 and anti-α-tubulin antibodies. NC65 and SW839 cells were seeded in high-glucose medium and then the culture medium was replaced on day 2 with fresh high-glucose medium (25 mM glucose) or with glucose-deprived medium (0 mM glucose) for 1–2 days. Two days after replacement of medium, both sets of cells were transferred into fresh medium containing glucose. (TIF) [file pone.0096168.s001.tif]

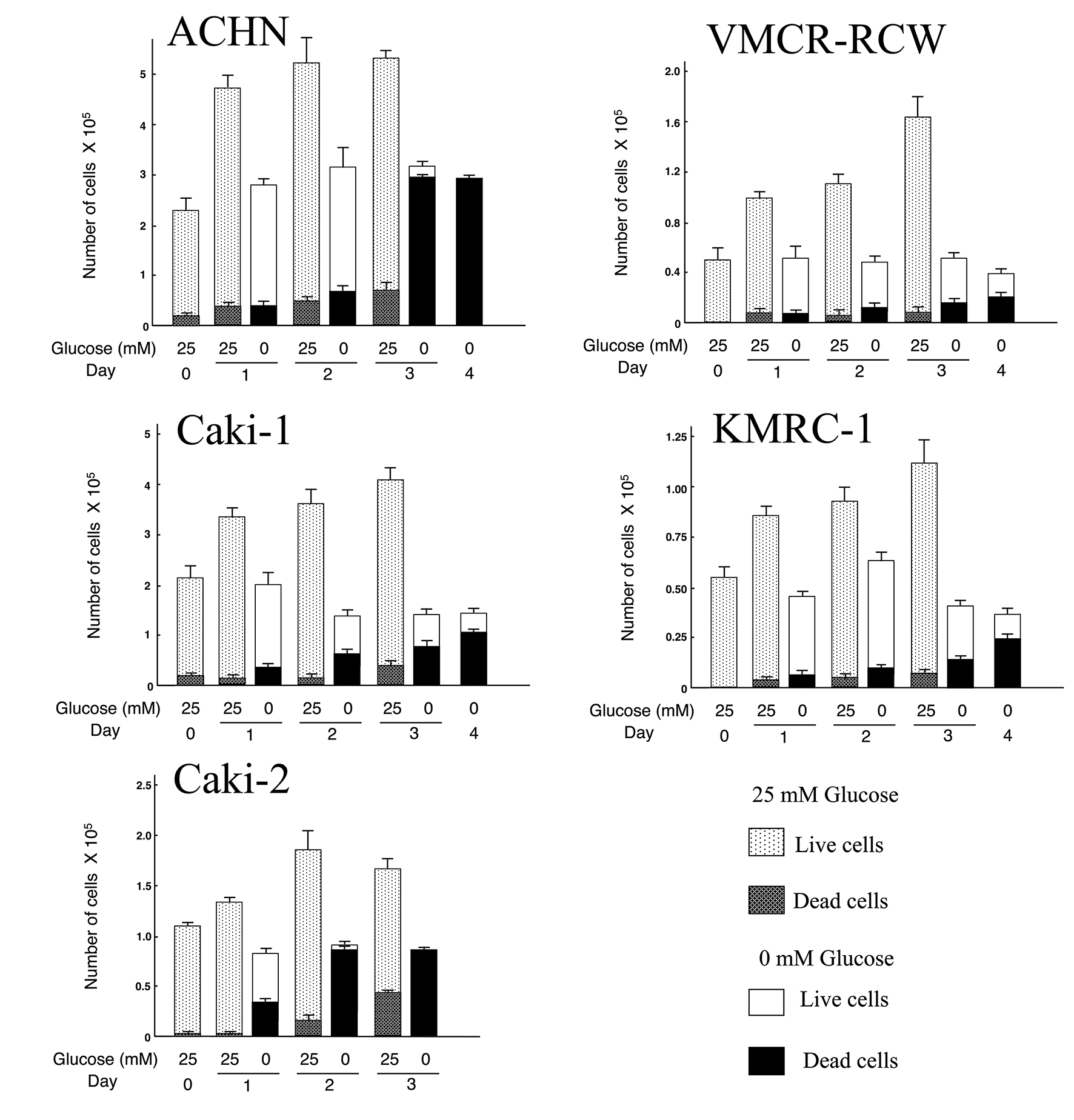

Supplement: Figure S2 — Cell growth under glucose deprivation in renal cell carcinomas. The numbers of living and dead cells were counted using the trypan-blue exclusion assay. Note that glucose deprivation significantly induced cell death in ACHN, Caki1 and Caki2 cells, but not in VMCR-RCW and KMRC-1 cells. (TIF) [file pone.0096168.s002.tif]

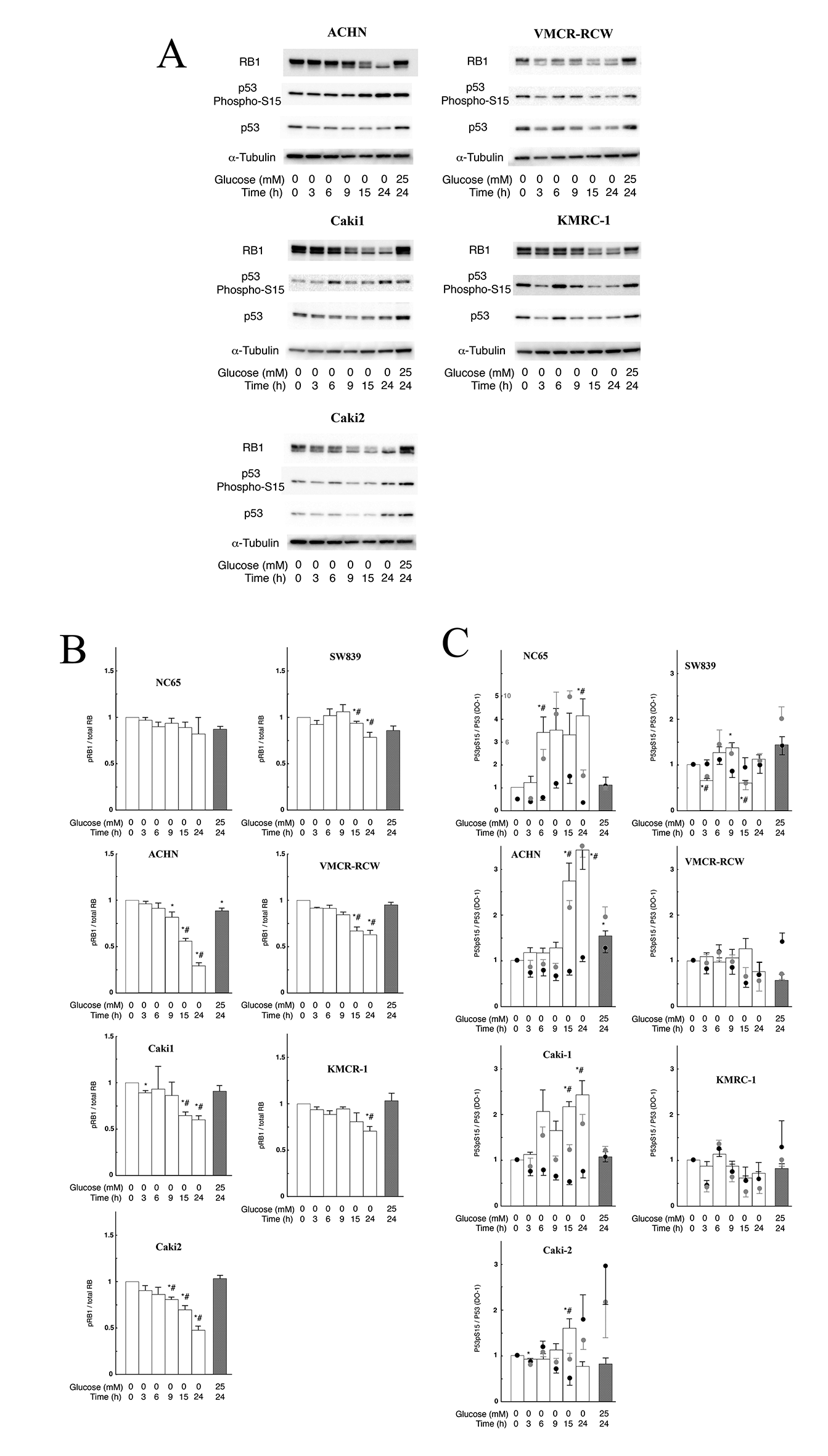

Supplement: Figure S3 — Immunoblot analysis of renal cell carcinomas. A, Immunoblots for RB1, S15-phosphorylated p53, total p53 and α-tubulin. B–C, Quantitative analysis of reactivity with the phosphorylated RB1 (pRB1) (B, upper bands), normalized to the total signal, and the S15-phosphorylated p53 (C), normalized to the total p53 signal in 0 mM glucose and 0 h, respectively. * and #: signify p<0.05 against 0 mM glucose at 0 h and 25 mM glucose at 24 h, respectively. Note that glucose deprivation significantly reduced the level of phosphorylated RB1 in ACHN, Caki1 and Caki2 cells. S15-phosphorylated p53 was induced under conditions of glucose deprivation in NC65, ACHN, Caki1 and Caki2 cells. (TIF) [file pone.0096168.s003.tif]

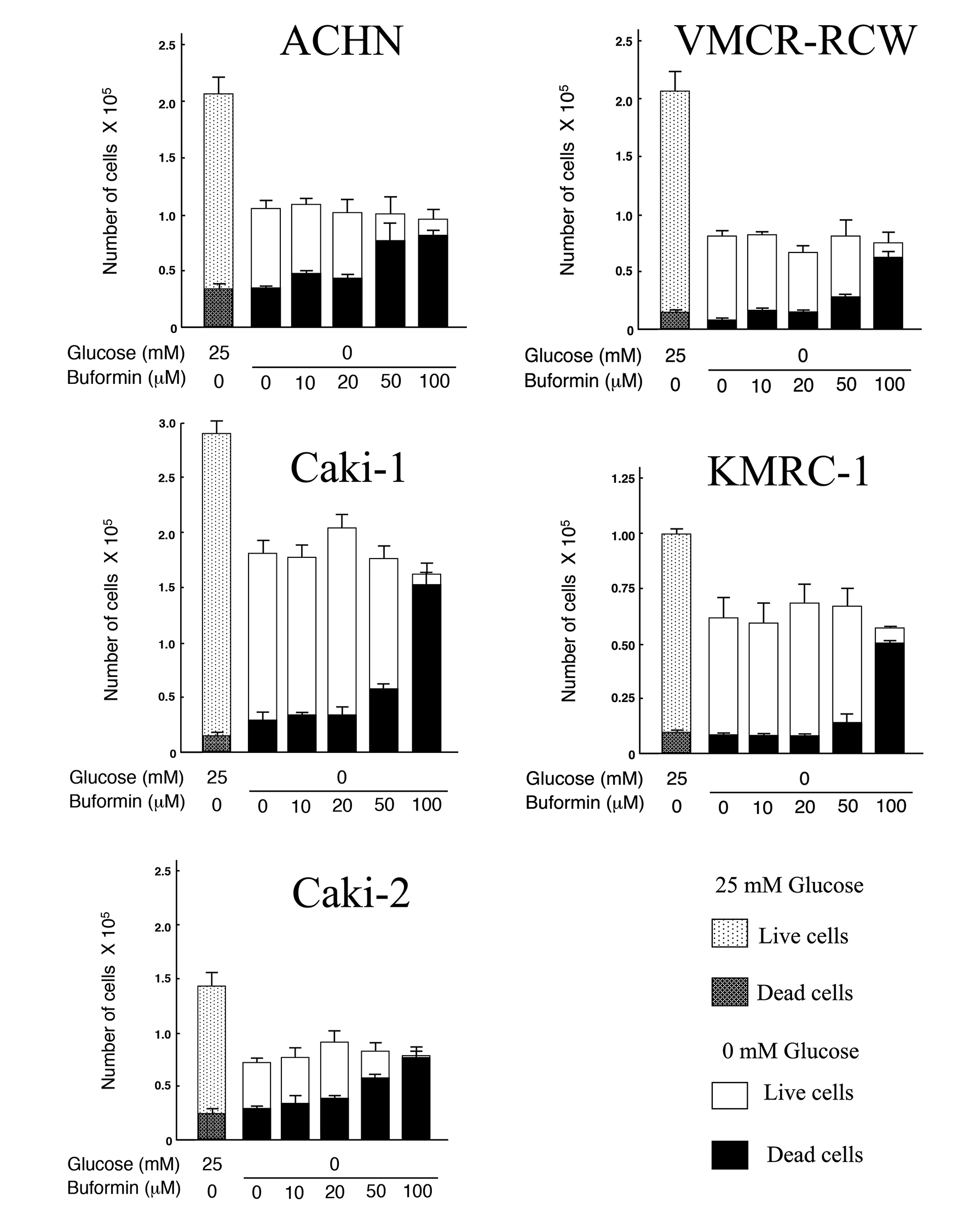

Supplement: Figure S4 — Buformin could induce cell death in all types of renal cell carcinomas under conditions of glucose deprivation. Renal cell carcinomas were cultured in 25 mM or 0 mM glucose medium with or without buformin for 24 h. The numbers of living and dead cells were counted using the trypan-blue exclusion assay. Note that buformin could induce significant levels of cell death in all types of renal cell carcinomas under glucose deprivation. (TIF) [file pone.0096168.s004.tif]
