# Supplementary material for: Glucose Deprivation Induces G2/M Transition-Arrest and Cell Death in N-GlcNAc2-Modified Protein-Producing Renal Carcinoma Cells
Source: PLoS One. 2014 May 5;9(5):e96168. doi: 10.1371/journal.pone.0096168 (PMC4010426; doi:10.1371/journal.pone.0096168)
Supplement: Table S1 — Oligonucleotides used for qRT-PCR. (DOC) [file pone.0096168.s005.doc]

**Table S1. Oligonucleotides used for qRT-PCR**

| Gene | Forward (5’ to 3’) | Reverse (5’ to 3’) |
| --- | --- | --- |
| *GFPT1*  *UAP1* | TACCATGTTCCTCGAACGAGAC  CAAAGGAGTTCTTCACCAAGCAC | TGCTTGTGAACTTCTTCATCCAG  AAAGACCACCATTCCCATCTGGAG |
| *CDKN1A* | GCGGCAGACCAGCATGACAG | CAGGGTATGTACATGAGGAG |
| *GADD45A* | GCGGCCAAGCTGCTCAACGTC | ACGCCTGGATCAGGGTGAAGTG |
| *ATF3*  Spliced *XBP1*  *GAPDH* | TGCCTGTCCCCTCCTGGGTC  TGAGTCCGCAGCAGGTGCAGG  GGGAGCCAAAAGGGTCATCATC | TCTTCTTCAGGGGCTACCTCG  ACATGACTGGGTCCAAGTTGTCC  TGGCATGGACTGTGGTCATGAG |
