# Supplementary material for: Glucose Deprivation Induces G2/M Transition-Arrest and Cell Death in N-GlcNAc2-Modified Protein-Producing Renal Carcinoma Cells
Source: PLoS One. 2014 May 5;9(5):e96168. doi: 10.1371/journal.pone.0096168 (PMC4010426; doi:10.1371/journal.pone.0096168)
Supplement: Table S2 — Flow cytometric analysis for renal cell carcinomas. (DOC) [file pone.0096168.s006.doc]

**Table S2. Flow cytometric analysis for renal cell carcinomas.**

|  | sub G1 | G1 | S | G2/M | MPM2 |
| --- | --- | --- | --- | --- | --- |
| NC65 +G | 1.4 | 53 | 12.7 | 32.3 | 31.7 |
| NC65 -G | **5.9** | **44.7** | **19.8** | 29 | **58.5** |
|  |  |  |  |  |  |
| ACHN +G | 2.3 | 34.4 | 17 | 45.5 | 39.7 |
| ACHN -G | **4.7** | **33.2** | **16.1** | 41.3 | **63.7** |
|  |  |  |  |  |  |
| Caki1+G | 1.6 | 62.9 | 7.4 | 27.3 | 26.3 |
| Caki1 -G | **6.2** | **54.5** | **12** | 26.6 | **61.7** |
|  |  |  |  |  |  |
| Caki2+G | 9.4 | 64.3 | 15.2 | 10.6 | 10.7 |
| Caki2 -G | **27.3** | **49.7** | **14.9** | 7.7 | **41** |
|  |  |  |  |  |  |
| SW839+G | 4.4 | 66 | 7.2 | 21.7 | 23.2 |
| SW839 -G | 3 | **68** | **5.7** | 23.1 | 26.3 |
|  |  |  |  |  |  |
| VMCR-RCW+G | 6.5 | 42.5 | 13.3 | 37.1 | 30.2 |
| VMCR-RCW -G | 8.7 | **48.6** | **5.5** | 36.1 | 37 |
|  |  |  |  |  |  |
| KMRC-1+G | 2.7 | 52.6 | 8.6 | 34.7 | 30.9 |
| KMCR-1 -G | 2.3 | **62.3** | **3.9** | 31.1 | 63.3 |

Renal cell carcinomas were cultured in fresh 25 mM or 0 mM glucose medium for 24h after 2 days of culture in 25 mM glucose medium and then fixed and stained using FxCycle^TM^ Violet nuclear staining reagent and anti-MPM-2 antibody. Scores of cell phases and MPM2-positive cells are showed a parcentage of total cells and avarage of duplicate or triplicate experiments. Bold show the mentioned scores in the text.
