# Supplementary material for: Glucose Deprivation Induces G2/M Transition-Arrest and Cell Death in N-GlcNAc2-Modified Protein-Producing Renal Carcinoma Cells
Source: PLoS One. 2014 May 5;9(5):e96168. doi: 10.1371/journal.pone.0096168 (PMC4010426; doi:10.1371/journal.pone.0096168)
Supplement: Table S3 — Quantitative RT-PCR data of GFPT1 and UAP1 belonging to the UDP-GlcNAc biosynthesis-pathway in renal cell carcinomas. (DOC) [file pone.0096168.s007.doc]

**Table S3. Quantitative RT-PCR data of *GFPT1* and *UAP1* belonging to the UDP-GlcNAc biosynthesis-pathway in renal cell carcinomas.**

| GFPT1 | 0mM 0h | 0mM 3h | 0mM 6h | 0mM 9h | 0mM 24h | 25mM 24h |
| --- | --- | --- | --- | --- | --- | --- |
| NC65 | 1.00 ± 0.15 | 1.40 ± 0.02 | *2.57 ± 0.17* | **3.66 ± 0.25** | **3.20 ± 0.06** | 1.19 ± 0.41 |
| ACHN | 1.00 ± 0.05 | *1.12± 0.03* | **1.95 ± 0.06** | **1.94 ± 0.09** | **3.51± 0.08** | 0.90 ± 0.04 |
| Caki1 | 1.00 ± 0.05 | 1.72 ± 0.57 | **2.92 ± 0.15** | **2.06 ± 0.18** | **2.17 ± 0.16** | *0.79 ± 0.04* |
| Caki2 | 1.00 ± 0.04 | **2.43 ± 0.13** | **5.24 ± 0.40** | **9.10± 0.35** | **5.86 ± 1.22** | 1.07 ± 0.16 |
|  |  |  |  |  |  |  |
| SW839 | 1.00 ± 0.19 | 1.56 ± 0.09 | 1.26 ± 0.17 | 1.39 ± 0.16 | *2.57 ± 0.44* | 1.49 ± 0.24 |
| VMCR-RCW | 1.00 ± 0.06 | 0.94 ± 0.23 | *1.58 ± 0.11* | *2.19 ± 0.12* | **4.35 ± 0.18** | 1.42 ± 0.41 |
| KMCR-1 | 1.00 ± 0.08 | 1.19 ± 0.03 | 1.15 ± 0.05 | 0.93 ± 0.07 | **2.28 ± 0.05** | 0.92 ± 0.03 |
|  |  |  |  |  |  |  |
| UAP1 | 0mM 0h | 0mM 3h | 0mM 6h | 0mM 9h | 0mM 24h | 25mM 24h |
| NC65 | 1.00 ± 0.14 | 0.99 ± 0.03 | 1.16 ± 0.03 | 1.26 ± 0.04 | 1.05 ± 0.01 | 0.92 ± 0.23 |
| ACHN | 1.00 ± 0.05 | **1.96 ± 0.27** | **4.21 ± 0.33** | **4.63 ± 0.44** | **5.56 ± 0.20** | 1.07± 0.03 |
| Caki1 | 1.00 ± 0.02 | 1.57 ± 0.26 | **6.08 ± 0.38** | **5.68 ± 0.38** | **4.47 ± 0.75** | *1.26± 0.09* |
| Caki2 | 1.00 ± 0.22 | 1.51 ± 0.17 | 4.06 ± 2.12 | **2.79 ± 0.20** | **2.62 ± 0.15** | 1.25± 0.11 |
|  |  |  |  |  |  |  |
| SW839 | 1.00 ± 0.15 | *1.64 ± 0.10* | 1.37 ± 0.08 | *1.86 ± 0.18* | 1.99 ± 0.40 | 1.36 ± 0.22 |
| VMCR-RCW | 1.00 ± 0.05 | 0.69 ± 0.41 | 1.59 ± 0.83 | 1.79 ± 0.49 | **3.30 ± 0.18** | 1.14 ± 0.10 |
| KMCR-1 | 1.00 ± 0.09 | *2.29 ± 0.33* | *1.94 ± 0.31* | 1.28 ± 0.09 | *1.93 ± 0.16* | 1.36 ± 0.15 |

Gene expression was normalized using the *GAPDH* gene and the expression of 0 mM glucose at 0 h. Results of experiments are represented as mean ± S.E. Each mean represents data from at three independent experiments. The Student’s *t* test (two-tail) was used to compare differences between groups. Bold signifies p < 0.05 against both 0 mM glucose at 0 h and 25 mM glucose at 24 h. Italic signifies p < 0.05 against 0 mM glucose at 0 h or 25 mM glucose at 24 h.
