# Supplementary material for: Glucose Deprivation Induces G2/M Transition-Arrest and Cell Death in N-GlcNAc2-Modified Protein-Producing Renal Carcinoma Cells
Source: PLoS One. 2014 May 5;9(5):e96168. doi: 10.1371/journal.pone.0096168 (PMC4010426; doi:10.1371/journal.pone.0096168)
Supplement: Table S4 — Quantitative RT-PCR data of GADD45A and CDKN1A belonging to the p53 signaling-pathway in renal cell carcinomas. (DOC) [file pone.0096168.s008.doc]

**Table S4. Quantitative RT-PCR data of *GADD45A* and *CDKN1A* belonging to the p53 signaling-pathway in renal cell carcinomas.**

| GADD45A | 0mM 0h | 0mM 3h | 0mM 6h | 0mM 9h | 0mM 24h | 25mM 24h |
| --- | --- | --- | --- | --- | --- | --- |
| NC65 | 1.00 ± 0.22 | **3.47 ± 0.28** | **12.52 ± 2.18** | **18.88 ± 5.27** | **16.56 ± 2.58** | 1.33 ± 0.16 |
| ACHN | 1.00 ± 0.06 | **2.39 ± 0.28** | **5.67 ± 0.30** | **4.39 ± 0.44** | **7.19 ± 0.68** | 1.14 ± 0.06 |
| Caki1 | 1.00 ± 0.39 | **8.46 ± 0.46** | **13.36 ± 0.82** | **9.42 ± 1.64** | **12.37 ± 1.45** | 0.71 ± 0.13 |
| Caki2 | 1.00 ± 0.09 | **16.64 ± 0.80** | **28.93 ± 1.84** | **27.74 ± 0.82** | **21.51 ± 1.04** | 1.04 ± 0.12 |
|  |  |  |  |  |  |  |
| SW839 | 1.00 ± 0.17 | **2.56 ± 0.40** | *1.23 ± 0.17* | **2.22 ± 0.82** | **3.26 ± 0.59** | 0.71 ± 0.07 |
| VMCR-RCW | 1.00 ± 0.05 | *2.35 ± 0.16* | **3.64 ± 0.50** | **7.11 ± 1.35** | **5.24 ± 0.22** | *1.73 ± 0.19* |
| KMCR-1 | 1.00 ± 0.06 | **2.99 ± 0.28** | **1.99 ± 0.27** | *1.12 ± 0.01* | **2.74 ± 0.19** | *0.63 ± 0.03* |
|  |  |  |  |  |  |  |
| CDKN1A | 0mM 0h | 0mM 3h | 0mM 6h | 0mM 9h | 0mM 24h | 25mM 24h |
| NC65 | 1.00 ± 0.05 | 1.62 ± 0.53 | 1.37± 0.18 | **2.21 ± 0.10** | **2.91 ± 0.23** | 1.10 ± 0.15 |
| ACHN | 1.00 ± 0.08 | *0.32 ± 0.17* | 0.58 ± 0.29 | 1.06 ± 0.09 | **2.08 ± 0.03** | 0.78± 0.07 |
| Caki1 | 1.00 ± 0.04 | *0.83 ± 0.01* | *1.07 ± 0.10* | 1.07 ± 0.10 | **2.43 ± 0.04** | *0.76 ± 0.03* |
| Caki2 | 1.00 ± 0.21 | **1.99 ± 0.16** | **2.70 ± 0.27** | **4.85 ± 0.07** | **6.15 ± 0.71** | 0.65 ± 0.13 |
|  |  |  |  |  |  |  |
| SW839 | 1.00 ± 0.05 | **3.91 ± 0.27** | **2.08 ± 0.02** | **2.02 ± 0.09** | **2.24 ± 0.23** | *1.46 ± 0.09* |
| VMCR-RCW | 1.00 ± 0.09 | **2.29 ± 0.04** | **2.72 ± 0.08** | **2.17 ± 019** | **2.63 ± 0.18** | 1.01 ± 0.00 |
| KMCR-1 | 1.00 ± 0.14 | 1.01 ± 0.12 | *1.29 ± 0.08* | 0.96 ± 0.05 | *1.33 ± 0.03* | 0.84 ± 0.09 |

Gene expression was normalized using the *GAPDH* gene and the expression of 0 mM glucose at 0 h. Results of experiments are represented as mean ± S.E. Each mean represents data from at three independent experiments. The Student’s *t* test (two-tail) was used to compare differences between groups. Bold signifies p < 0.05 against both 0 mM glucose at 0 h and 25 mM glucose at 24 h. Italic signifies p < 0.05 against 0 mM glucose at 0 h or 25 mM glucose at 24 h.
