# Supplementary material for: Glucose Deprivation Induces G2/M Transition-Arrest and Cell Death in N-GlcNAc2-Modified Protein-Producing Renal Carcinoma Cells
Source: PLoS One. 2014 May 5;9(5):e96168. doi: 10.1371/journal.pone.0096168 (PMC4010426; doi:10.1371/journal.pone.0096168)
Supplement: Table S5 — Quantitative RT-PCR data of ATF3 and XBP1 belonging to the UPR genes in renal cell carcinomas. (DOC) [file pone.0096168.s009.doc]

**Table S5. Quantitative RT-PCR data of *ATF3* and *XBP1* belonging to the UPR genes in renal cell carcinomas.**

| ATF3 | 0h | 0mM 3h | 0mM 6h | 0mM 9h | 0mM 24h | 25mM 24h |
| --- | --- | --- | --- | --- | --- | --- |
| NC65 | 1.00 ± 0.09 | **3.65 ± 0.08** | **5.41 ± 0.07** | **7.93 ± 1.65** | **8.61 ± 0.24** | 1.17 ± 0.11 |
| ACHN | 1.00 ± 0.12 | **1.99± 0.04** | **2.77 ± 0.10** | **2.89 ± 0.10** | **9.98 ± 0.44** | 1.06 ± 0.09 |
| Caki1 | 1.00 ± 0.03 | **8.39 ± 0.87** | **11.21 ± 0.92** | **9.23 ± 0.52** | **23.94 ± 0.76** | *1.28 ± 0.08* |
| Caki2 | 1.00 ± 0.29 | **7.50 ± 0.60** | **13.16 ± 0.83** | **9.10± 0.37** | **12.76 ± 1.00** | 0.80 ± 0.04 |
|  |  |  |  |  |  |  |
| SW839 | 1.00 ± 0.04 | **8.32 ± 0.83** | **1.23 ± 0.03** | **2.24 ± 0.08** | **4.07 ± 0.18** | 0.77 ± 0.02 |
| VMCR-RCW | 1.00 ± 0.02 | **3.54 ± 0.19** | **3.05 ± 0.14** | **4.14 ± 0.15** | **7.62 ± 0.23** | 1.09 ± 0.02 |
| KMCR-1 | 1.00 ± 0.16 | **4.44 ± 0.16** | **2.29± 0.10** | *1.31 ± 0.09* | **8.48 ± 0.32** | 0.67 ± 0.04 |
|  |  |  |  |  |  |  |
| Spliced XBP1 | 0h | 0mM 3h | 0mM 6h | 0mM 9h | 0mM 24h | 25mM 24h |
| NC65 | 1.00 ± 0.09 | **1.86 ± 0.07** | **3.31 ± 0.08** | **3.32 ± 0.10** | **3.88 ± 0.43** | 1.05 ± 0.10 |
| ACHN | 1.00 ± 0.02 | **1.93± 0.02** | **4.36 ± 0.08** | **4.10 ± 0.08** | **5.73 ± 0.35** | *1.23 ± 0.04* |
| Caki1 | 1.00 ± 0.07 | **2.59 ± 0.10** | **3.27 ± 0.14** | **2.10 ± 0.22** | **2.42 ± 0.08** | 0.61 ± 0.12 |
| Caki2 | 1.00 ± 0.02 | **3.26 ± 0.12** | **5.24 ± 0.10** | **5.50 ± 0.06** | **5.81 ± 0.08** | 0.99 ± 0.02 |
|  |  |  |  |  |  |  |
| SW839 | 1.00 ± 0.04 | **2.44 ± 0.05** | **1.36 ± 0.06** | **2.20 ± 0.06** | **3.28 ± 0.16** | 1.00 ± 0.03 |
| VMCR-RCW | 1.00 ± 0.02 | **2.00 ± 0.02** | **2.17 ± 0.18** | **2.32 ± 0.04** | **3.00 ± 0.02** | 1.11 ± 0.05 |
| KMCR-1 | 1.00 ± 0.02 | **1.52 ± 0.01** | **1.29 ± 0.01** | **2.01 ± 0.06** | **3.56 ± 0.04** | *1.45 ± 0.02* |

Gene expression was normalized using the *GAPDH* gene and the expression of 0 mM glucose at 0 h. Results of experiments are represented as mean ± S.E. Each mean represents data from at three independent experiments. The Student’s *t* test (two-tail) was used to compare differences between groups. Bold signifies p < 0.05 against both 0 mM glucose at 0 h and 25 mM glucose at 24 h. Italic signifies p < 0.05 against 0 mM glucose at 0 h or 25 mM glucose at 24 h.
